# Implementing electronic informed consent in rare disease genomics

**DOI:** 10.1038/s41598-025-32740-1

**Published:** 2025-12-23

**Authors:** Katja Ekholm, Annelie Augustinsson, Johan Sundström, Christian Johansen, Maria Storgärds, Lena Ljöstad, Fulya Taylan, Eva Ekblom, Stephanie Juran, Marlene Ek, Charlotta Ingvoldstad Malmgren, Maria Johansson Soller, Mikaela Friedman, Sofia Thunström, Lovisa Lovmar, Ann Nordgren, Hans Ehrencrona, Anna Lindstrand

**Affiliations:** 1 Department of Clinical Genetics and Genomics, Karolinska University Hospital, Stockholm, Sweden; 2 Department of Molecular Medicine and Surgery, Karolinska Institutet, Stockholm, Sweden; 3 Care in High Technological Environments, Department of Health Sciences, Lund University, Lund, Sweden; 4 Department of Clinical Genetics, Pathology and Molecular Diagnostics, Skåne University Hospital, Lund, Sweden; 5 Department of Medical Sciences, Clinical Epidemiology, Uppsala University, Uppsala, Sweden; 6 MinForskning AB, Uppsala, Sweden; 7 Rare Diseases Sweden, National alliance for people living with rare diseases, Sundbyberg, Sweden; 8 Department of Clinical Genetics and Genomics, Sahlgrenska University Hospital, Region Vastra Gotaland, Gothenburg, Sweden; 9 Department of Laboratory Medicine, Institute of Biomedicine, Sahlgrenska Academy at University of Gothenburg, Gothenburg, Sweden; 10 Division of Clinical Genetics, Department of Laboratory Medicine, Lund University, Lund, Sweden

**Keywords:** Electronic informed consent (eConsent), Whole-genome sequencing, Rare diseases, Genomics, Computational biology and bioinformatics, Genetics, Health care, Medical research

## Abstract

In rare disease research, sharing of individual health data is essential for advancing diagnostics and therapies, requiring robust and ethically sound informed consent processes. Within the Genomic Medicine Sweden Rare Diseases (GMS-RD) multicenter study, an electronic informed consent (eConsent) platform was developed to support data sharing, facilitate participation and enable research engagement. Tailored to the complex consent needs of rare disease contexts, the platform was piloted at three Genomic Medicine Centers in Sweden. A total of 2244 individuals were invited in the clinical routine cohort, with an overall eConsent rate of 18.6%. Uptake was highest among adult singletons (27.8%) and lowest in trios (14.3%). In contrast, the Undiagnosed Diseases Network (UDN) Sweden cohort achieved a 94% consent rate, attributed to targeted communication and active patient organization involvement. Key challenges included technical accessibility limitations, digital literacy deficits, comprehension and language barriers, and the burden of multi-step processes, especially for families. Findings underscore the need to improve usability, strengthen communication, and implement flexible consent management over time. eConsent can broaden participation in genomic research and promote responsible data sharing. However, in rare diseases contexts, it must be designed with inclusivity, clarity, and adaptability to meet the diverse participant needs.

**Supplementary Information:**

The online version contains supplementary material available at 10.1038/s41598-025-32740-1.

## Background

Each rare disease affects fewer than one in 2000 individuals, and approximately 72% are thought to have a genetic origin^1,2^. The introduction of massive parallel sequencing has revolutionized rare disease diagnostics, enabling clinical exome and genome analysis with overall diagnostic yields ranging from 20 to 50%, depending on inclusion criteria (reviewed in^3^). Despite these advances, many individuals continue to face prolonged diagnostic odysseys. Even with comprehensive approaches such as whole genome sequencing (WGS), more than 60% of cases remain undiagnosed^3^. This diagnostic gap is due in part to incomplete phenotypic information, the challenge of interpreting variants of uncertain significance (VUS), and current technical, as well as knowledge-based, limitations in detecting the full spectrum of disease-causing variants. Given the small number of individuals affected by each specific condition, data sharing across centers and networks is crucial. By aggregating individual genomic and clinical data, researchers and clinicians can improve variant interpretation, uncover disease mechanisms, and accelerate progress toward precise diagnostics and personalized care and treatments. Importantly, there is an intimate relationship between clinical diagnostics and research in rare diseases, where new discoveries often emerge from studying individual cases. The active involvement of individuals living with rare diseases in research initiatives is essential, not only to improve diagnostic and therapeutic care at the individual level, but also to advance collective knowledge through responsible data sharing and collaboration^4^.

In the context of electronic informed consent for studies where participants consent to the secondary use of biological samples and genomic data, several binding legal frameworks apply. Within the European Union, the General Data Protection Regulation (GDPR)^5^ provides the overarching legal basis for processing sensitive personal data, including health and genomic information, and sets out the requirements for explicit and informed consent. Additional provisions relevant to biomedical research are set out in the EU Clinical Trials Regulation^6^ while secure and standardized data sharing is further supported by the European Data Governance Act^7^. In Sweden, research involving human subjects, genetic data, and the secondary use of biological samples is regulated by the Act concerning the Ethical Review of Research Involving Humans^8^ and the Swedish Biobank Act^9^, which govern the collection, storage and reuse of human biological material for research purposes.

Although there is no dedicated legislation that specifically regulates electronic informed consent, these frameworks together govern the legal requirements for consent procedures (including electronic consent) and for the lawful processing, storage and sharing of genetic and health-related data, including consent for secondary use.

Informed consent is a fundamental principle in both research and medical practice, designed to safeguard participants’ autonomy by ensuring that they voluntarily participate with a clear understanding of the associated risks, benefits, and procedures. The American Psychological Association (APA) defines informed consent as “the process by which researchers explain their study to human participants and obtain their approval based on their understanding of the research’s methods and objectives”^10^. The Declaration of Helsinki emphasizes that voluntary informed consent must be obtained before enrolling participants in research^11^. Similarly, the Council for International Organizations of Medical Sciences (CIOMS) highlights the importance of respecting participants’ autonomy and their right to withdraw at any time without negative consequences^12^.

In the context of genetic and genomic research, informed consent must address specific considerations such as the inability to make data anonymous, possibility of incidental findings, implications for biological relatives, long-term data storage, and future secondary use of genomic data. Participants should also be informed about the potential benefits, such as receiving a diagnosis or contributing to the development of targeted therapies. Given the sensitive and enduring nature of genomic information, clear communication about data privacy and sharing is essential^13^.

Informed consent has traditionally relied on paper-based processes, but electronic consent (eConsent) platforms are creating new opportunities for participant engagement. eConsent offers a flexible, participant-centered approach that uses interactive and digital tools to enhance understanding^14,15^. This innovation is particularly relevant in an era of growing demands for transparency, faster research cycles, and remote or decentralized study designs^16^. Participant comprehension is a known challenge in consent procedures, and integrating clear communication strategies and interactive elements into eConsent platforms may help address this issue^17,18^. Furthermore, dynamic consents, a more flexible, patient-centered model, can foster long-term engagement and accommodate evolving preferences and study needs^19^. However, it is important to acknowledge that some individuals may face difficulties navigating electronic systems^20^ or may lack access to digital tools, emphasizing the need for accessible and inclusive design in eConsent implementation.

Genetic data is particularly sensitive due to its uniquely identifiable nature, its potential to reveal disease predispositions, and its implications not only for the individual but also for their biological relatives. As the reuse of genetic data becomes more common in research, concerns about privacy and autonomy have grown, highlighting the importance of broad consent frameworks and established ethical guidelines in safeguarding participants. Such frameworks are essential not only to protect participants but also to ensure the continued trust of patients, demonstrating our commitment to safeguarding their data and privacy as we share and utilize genetic information^21,22^. Nevertheless, people living with rare diseases have generally shown a high willingness to share their genetic data^4^.

Genomic Medicine Sweden (GMS), initiated in 2017, aims to integrate genomics-based diagnostics in routine healthcare and build a sustainable national infrastructure for precision medicine^23,24^. As of 2025,WGS is offered as a clinical diagnostic test at four of Sweden’s seven university hospital laboratories, with more than 30,000 individuals tested within the Swedish healthcare system^25^.

In this article, we describe our experiences with informing and consenting individuals with rare diseases, who face diverse medical, cognitive, or practical challenges, using an electronic consent process. We focus on the benefits and challenges encountered during implementation and highlight the importance of flexible systems that support participant understanding, trust, and informed decision-making regardless of individual abilities, circumstances, or mode of consent.

## Methods

### GMS-RD project and ethical approval

The national multicenter project “GMS-RD: Identification of Novel Causes of Rare Genetic Diseases” was registered and approved by the Swedish Ethical Review Authority in December 2019 (Ref no 2019-04746). In brief, the study allows for clinical, functional and psychosocial studies of patients as well as sharing of genomic data with national and international research initiatives such as the “Beyond 1 Million Genomes” initiative^26^ and the European Health Data Space^27^. The study is the foundation of national data sharing within GMS-RD where data will be stored in the National Genomics Platform (NGP), a high compute cluster that enables both storage and computation^23^.

Swedish healthcare has historically relied on paper-based informed consents, but an eConsent system was introduced to facilitate data sharing and research participation in the era of genomic medicine. The eConsent platform, minforskning.se, was further developed and tailored to the specific needs of the GMS-RD project through a collaboration between GMS and Uppsala University. The platform implementation commenced with the first participant enrollment on September 27, 2023.

### Inclusion sites and clinical cohorts

The eConsent platform was piloted in a clinical routine setting at three Genomic Medicine Centers (GMC) in Sweden located at Sahlgrenska University Hospital (GMC West, Gothenburg), Karolinska University Hospital (GMC-K, Stockholm) and Skåne University Hospital (GMC South, Lund). All sites offer WGS as part of their clinical genetic diagnostics programs and analyses are performed as both trios and singletons and both with panel and whole-genome interpretation. eConsent evaluation included trio consent cohorts (affected child with both parents having also analyzed samples) and singleton consent cases (affected child or adult). eConsent was also separately evaluated for the nationwide Undiagnosed Diseases Network Sweden (UDN Sweden) initiative, which includes trio sample investigations of individuals with intellectual disability (ID) or ID syndromes who remain undiagnosed after short-read WGS.

Although children were invited to participate in the study from infancy to 17 years of age, the individual consent form did not include a separate document for minors. Instead, legal guardians provided consent on behalf of their children in accordance with Swedish legislation on research involving minors^8^. The general study description, available on the electronic platform, includes information leaflets adapted to minor assent, with separate wordings for children (6–10 years) and young people (11–17 years). Moreover, two brief animated videos adapted for a younger audience are available on the platform, explaining the concepts of whole genome sequencing and precision medicine.

### Technical solution for an electronic consent database and data collection

We used the solution minforskning.se, originally developed as part of a research study at Uppsala University, but later commercialized in the company MinForskning AB (Uppsala, Sweden)^28–30^. The system has been used in seven additional studies, including studies with and without the co-signature of a physician, studies where participants are recruited via social media without the need for a co-signature, clinical drug trials requiring a physician’s co-signature and, one study where the legal guardians provide consent on behalf of their minor children. Minforskning.se provides public access only to general study information and contact details for the study team. Access to any personal information, including individual consent forms, requires secure login with BankID, the largest Swedish electronic identification system, that has a usage rate of 94% among smartphone users in Sweden^31^ and is an accepted identification solution by national authorities. In minforskning.se, personal data are therefore accessible only after authentication with BankID, in accordance with GDPR requirements^5^. BankID is administered by Finansiell ID-Teknik BID AB, which is owned by several Swedish and Scandinavian banks. With BankID, participants can view and download signed eConsents and access withdrawal instructions. Anyone can create a profile to receive study offers and studies can send secure, individualized messages through the platform. Participants can independently consent to studies, or, for studies requiring a physician’s co-signature, the system ensures that signatures occur in the correct sequence in the same session. Through real-time checks with the Swedish Population Register, the system allows legal guardians to consent for minors, and mandates all (one or two) guardians to consent before an eConsent is considered valid. The system tracks eConsent status in real-time, and manual paper consents can be recorded to obtain a complete eConsent database for a study. In addition, an Application Programming Interface (API) solution makes it possible to ask questions about valid eConsents from other authorized systems, such as genomic databases. The platform complies with key international standards for clinical research and electronic data management. These include the Good Automated Manufacturing Practice 5 (GAMP 5)^32^, the International Council for Harmonisation Guideline for Good Clinical Practice E6 (ICH E6), the international Good Clinical Practice (GCP) guideline for clinical trials involving human participants^33^, the U.S. Food and Drug Administration Code of Federal Regulations (FDA CRF) 21 Part 11, which defines the use of electronic records and electronic signatures in FDA-regulated environments^34^, and EudraLex Annex 11, outlining the European Union’s requirements for computerized systems in pharmaceutical manufacturing and clinical trials^35^. All personal data is securely stored in Sweden in compliance with the General Data Protection Regulation (GDPR), with MinForskning AB acting as the designated data processor. Registered patient consents and other study-specific data is owned by the respective research institutions, while MinForskning AB holds intellectual property rights for the technical platform.

### General eConsent process

Individuals were invited to participate in the GMS-RD study through a letter in Swedish containing brief information about the study, the eConsent platform, and practical steps for consenting (examples of letter templates used translated to English are available as Supplementary materials (Additional file 1: Document S1)). Invited individuals were eligible to participate if they had undergone clinical WGS analysis at one of the participating Genomic Medicine Centers. No prior consent for research recontact was collected during the clinical procedure, instead, recontact occurred under the ethics approval of the GMS-RD research project, approved by the Swedish Ethical Review Authority. Access to the eConsent platform additionally required the ability to read Swedish and the use of a digital identification solution. However, individuals without digital ID were not excluded from participation. These participants were offered paper-based consent forms and could receive assistance from study personnel by phone or email if needed. All paper-based consents were subsequently entered into the eConsent database to ensure complete documentation and traceability for future research.

**Fig. 1:**
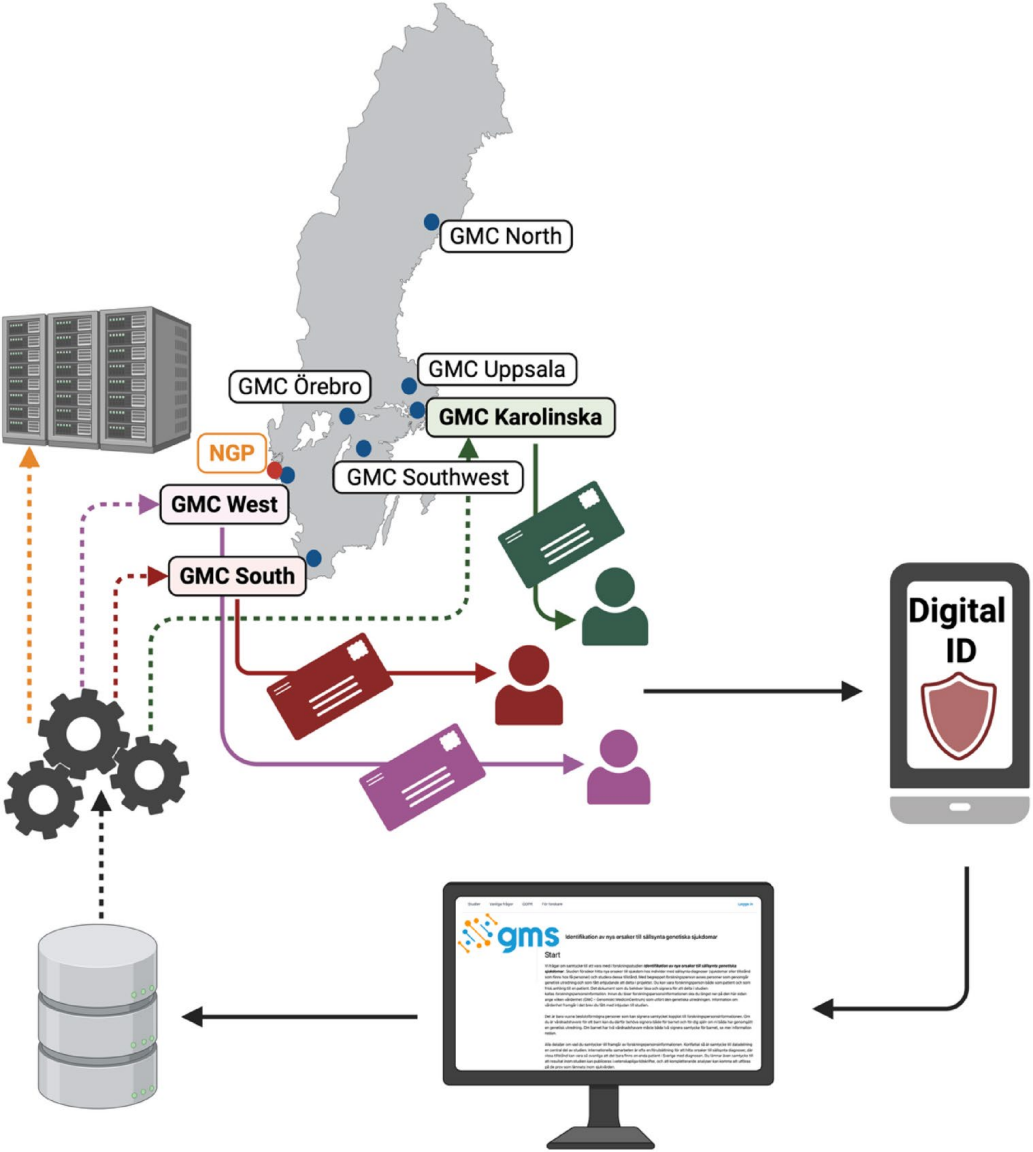
eConsent platform and consent process. Each Genomic Medicine Center (GMC) functions as a study site with its own portal entry. During the pilot phase, only GMC South (red arrow), GMC West (pink arrow), and GMC Karolinska (green arrow) were active. Invited potential participants received a letter with instructions for logging in using a digital ID, reviewing the Participant Information Sheet, and providing consent to participate. The signed consents were stored in a database accessible to both the GMCs and the National Genomic Data Platform (NGP). Created with BioRender.com.

In brief, for a singleton adult case, the process includes creation of a profile, choosing a study site, reading the informed consent and then signing with an electronic ID (BankID) (Fig. 1). For a singleton pediatric case, legal guardians (one or two) need to sign for the child. For a pediatric trio case, a complete trio consent is needed, where both biological parents need to sign for the child and also can choose to separately sign for themselves since their samples also has been collected. Individuals not speaking Swedish or lacking Bank ID could only join the study through standard paper-based alternatives, with the assistance of study personnel.

### Experiences and questions

Data were derived from participant inquiries and researcher notes, focusing on the practical and logistical challenges encountered during the consent process.

## Results

### Cohorts evaluated for eConsent

For the clinical cohort in total, 709 adult singletons, 448 pediatric singletons, and 1087 individuals belonging to trios (369 affected individuals and 718 healthy relatives, most often parents) were invited by letter to participate by registering eConsent in the GMS-RD study. Consents were obtained for 197 adult singletons, 65 pediatric singletons, and 61 trios (155 individuals), respectively. In average, this represented a consent rate of 18,6%, albeit with large differences between subgroups. For the UDN cohort the corresponding consent rate was 94%. (Table 1).

**Table 1:** Consent evaluation clinical cohorts and acceptance rates.

Cohort	Cohort type*	Site	Approached individuals	eConsented individuals	eConsent rate (%)
Clinical cohorts	Trio	GMC South	420	72	17.1%
	Trio	GMC Karolinska	120	3	2.5%
	Trio	GMC West	547	80	14.6%
	Singleton pediatric	GMC South	248	38	15.3%
	Singleton pediatric	GMC West	200	27	13.5%
	Singleton adult	GMC South	226	62	27.4%
	Singleton adult	GMC Karolinska	340	105	30.9%
	Singleton adult	GMC West	143	30	21.0%
UDN	Trio	UDN Sweden	303	285	94.0%

*For trios, consent was obtained from the child as well as from each parent, resulting in three individual consents in most families.

### Experiences from the national UDN Sweden study

The 101 children with undiagnosed rare ID and ID syndromes that met the inclusion criteria for the national UDN Sweden project had already spent years in the diagnostic odyssey. They were recruited mainly through patient organizations for undiagnosed individuals with rare diseases (Wilhelm Foundation and Anonymous) which spread information about the study as well as about the eConsent platform. Several information sessions were held where families also had the possibility to ask questions. In the end, digital consents were obtained from 94% trios (95/101 trios).

### Registration of paper consents in the eConsent database

In addition to primary electronic consents, a total of 2178 paper consents (2100 from Lund, 76 from Stockholm, 2 from Gothenburg) were registered retrospectively by study personnel in the eConsent database. These registrations are excluded from the above reported dataset.

### Participant experiences

In the different groups, data from participant inquiries and researcher notes, focusing on the practical and logistical challenges encountered during the consent process was compiled.

Letters describing the GMS-RD study and the eConsent platform were sent to 40 pediatric trio families in the GMC Karolinska cohort. Four families responded electronically, but only one successfully completed the digital consent for all family members. The remaining families either provided consent for only one parent and the child, or, in one case, for the parents only. Parents reported that the trio process was time-consuming and burdensome, as each parent or legal guardian was required to create separate accounts for themselves and for each child. Based on this feedback, we adopted a different approach for subsequent outreach, which was not included in the overall statistics for the initial pilot. In this adapted approach, 210 additional families were contacted by phone and offered the option to provide paper-based consent in addition to using the eConsent platform. Of note, among the families contacted using this modified strategy, all who chose to participate provided consent on paper. Many families called back multiple times, as one parent often wished to discuss participation with the other before making a decision. In several cases, families requested a callback the following day after internal discussions. In total, 148 of the 250 families opted to participate (60%).

The uptake of correct electronic consent in the GMC South pediatric (trio and singleton) cohort was considerably higher than for GMC Karolinska (17.1% and 15.3%, respectively). Almost all these families handled the registration without problem. One participant sent an email to ask if they had managed to register the consent for all three individuals in the trio, but in this case, everything was already in order. In addition, six emails and five phone calls were registered where the legal guardian had general questions about the project, but none of these questions were related specifically to the consenting process on the eConsent platform.

The uptake in the GMC West cohorts was similar to GMC South for the singleton pediatric and trio cohort. All three sites had higher up-take for the adult cohorts (21,0%, 27,4% and 30,4% for GMC West, GMC South and GMC Karolinska respectively). The GMC West cohort consisted almost exclusively of participants with a negative genetic screening result and hence differed in composition compared to the other sites. The most frequent reason of correspondence and questions raised at this site (19 contacts) was about the offer of study inclusion in it-self (9 contacts) rather than problems with the eConsent platform (4 contacts).

### Phone calls, e-mails and experiences

In all clinical cohorts, incoming questions from potential research participants were monitored following the distribution of invitation letters. In the GMC South singleton adult cohort (226 individuals approached), one email and five phone calls were received. In the GMC Karolinska adult cohort (340 individuals approached), 25 phone calls were received, and in the Sahlgrenska cohort (890 individuals approached), 19 emails were received. Most inquiries related to difficulties navigating the eConsent platform or clarifying the reason for inclusion. In one case, a letter had been sent to a deceased individual, underscoring the importance of cross-referencing participant lists with national registries to prevent distress to families. The following challenges were reported:

#### Login difficulties

Eighteen individuals had difficulties logging into the platform. Of note, in total nine elderly participants struggled with the eConsent platform and frequently required additional guidance. Two individuals from Stockholm and two from Lund lacked a digital identity verification system such as Bank-ID and could therefore not login to the system.

#### Information gaps

Other questions showed confusion about the purpose of the project (n = 15), the need for additional sample collection (n = 3) as well as concerns regarding the duration of consent validity but also positive feedback about the possibility to contribute to research and requests for inclusion.

Miscommunication led some participants to incorrectly believe they needed to provide new samples. We received feedback on information clarity and suggestions of allowing preferences for manual (paper-based) or oral consent (i.e., giving consent verbally rather than in writing).

A specific challenge is the inclusion of patients with a considerable time span between the performed analysis and the study inclusion, especially with negative results. They might not remember the performed analysis and hence question the offer to be included.

## Discussion

**Fig. 2:**
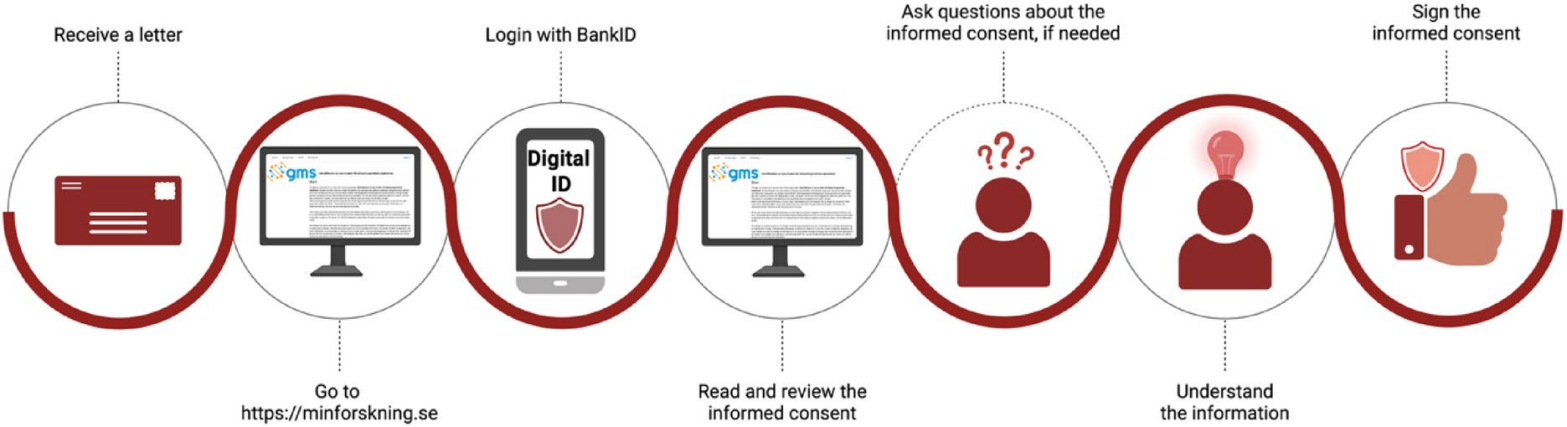
The GMS-RD eConsent experience.

This study outlines the experiences of GMS-RD in optimizing participant inclusion in a national multicenter rare disease project through the implementation of an electronic informed consent solution (eConsent) in addition to traditional paper-based consent (Fig. 2). Our findings indicate that the eConsent platform was more effective and practical for adult singleton cases than for pediatric scenarios (both singletons and trios), where additional logistical and communication challenges were encountered.

In rare disease research, data sharing is essential for fostering collaboration and accelerating discovery. Because rare diseases affect small and often dispersed populations, research findings are more valuable when shared across institutions and borders. Effective data sharing maximizes the utility of limited patient data, enabling researchers to identify patterns, refine diagnostic criteria, and advance treatment development, ultimately improving healthcare and quality of life for people living with rare diseases. Aggregating genetic and clinical data through shared resources also enables insights that would be difficult to achieve from smaller, isolated studies. However, the collection and sharing of sensitive health data raise important ethical and privacy concerns. It is essential that informed consent processes are robust and transparent, especially when data sharing is involved. Participants in rare disease studies must clearly understand how their data will be used, stored, and shared, with strong safeguards to protect their privacy. They should also be made aware of their right to withdraw from data-sharing initiatives at any time without negative consequences. Consent models that allow participants to manage and update their preferences over time, such as dynamic consent, offer a promising approach to addressing these concerns^36^.

In several countries, informed consent for genetic and genomic testing is often obtained in face-to-face consultation with a genetic counsellor or other trained genetics professional. In the Swedish context, clinical WGS for rare diseases is typically introduced within the routine healthcare by a responsible clinician, usually in collaboration with clinical geneticists and/or genetic counsellors, before diagnostic testing is performed. The eConsent process described in this study was intended for secondary research use and data sharing with GMS-RD, and was not designed to replace the diagnostic counselling. Instead, it was designed as an additional layer to existing clinical consent pathways, enabling participants to make an informed decision about the research use and long-term sharing of their genomic and clinical data.

In the clinical cohorts, 28% (197/709) of adult singletons provided eConsent, compared to 14% (220/1535) of pediatric patients and parents (Table 1). Although uptake was highest among adult singletons and lowest among pediatric trios, specific barriers differed between groups. Among elderly singleton participants, the most common challenges were login difficulties and low digital literacy, whereas in the pediatric trio scenario, the most frequently raised concern was the requirement for multiple guardian signatures to authorize participation. We predict that, even with an improved system, both of these scenarios will likely continue to benefit from personal guidance and support from trained personnel. Importantly, both the letters and the approved informed consent form were available only in Swedish, which limited inclusion of individuals with other language backgrounds.

The consent rate for trios was considerably higher in the GMC South (17.1%) and GMC West (14.6%) cohorts than in the GMC Karolinska cohort (2.5%), despite similar procedures for contacting the families, and the reason for this may be a subject for future studies. It should also be noted that the rate of acceptance in the national UDN study was much higher than for any of the other subgroups (95/101, 94%), probably due to the fact that the families in the UDN Sweden are highly motivated and typically have been actively searching for a diagnosis for years, often involving numerous contacts and discussions with healthcare providers, and also that patient associations were involved in the recruitment of these families and could provide support.

Although our study was not designed to capture sociodemographic information, it is possible that socioeconomic factors contributed to the variation in consent uptake between centers. Digital literacy, access to digital devices, language proficiency, and trust in digital platforms are known to vary across socioeconomic groups, potentially influencing the willingness or ability to use an electronic consent system. Without systematic data collection, these effects cannot be assessed in the present study. Therefore, future implementations should consider including sociodemographic variables and develop strategies to provide support, to ensure that electronic consent solutions do not unintentionally reinforce existing inequities in access to rare disease research.

Improved communication may also enhance participation, especially in complex cases like pediatric studies. Clear and accessible materials that explain the study’s purpose, procedures, and benefits in simple terms help both participants and guardians better understand their role. In rare disease research, where medical concepts can be challenging, tailoring language and format to the audience is crucial. Future development of the eConsent platform should therefore focus not only on technical usability but also on improving information clarity.

Furthermore, for an adult population the eConsent platform provides an unprecedented possibility to join advanced rare disease research, where this study indicates that acceptance rates of about 30% may be expected after sending an invitation letter, minimizing the burden on the healthcare system. Additionally, the eConsent enables broad recruitment without geographic and disease group bias. The possibility to manually add paper-based informed consents to the secure eConsent database is an important functionality, since consent queries over API will then be able to cover all participants in a study, regardless of the mode of consent registration.

This pilot implementation has several limitations. First, individuals without a digital ID solution, for example due to digital illiteracy, personal preference, or structural barriers, could not use the platform. However, these participants were not prevented from enrolling in the study since paper-based consent was offered as an alternative, and study personnel were available to provide support by phone or email. All paper consents were subsequently entered into the eConsent database. Nevertheless, the dependence on BankID for digital access should be recognized as a structural limitation of the current system. Second, the platform and invitation letters were available only in Swedish during the study period, which is likely to limit participation among individuals with limited proficiency in Swedish. Third, we did not systematically collect detailed sociodemographic data, which restricts our ability to fully analyze the impact of socioeconomic factors on consent uptake across the centres. These limitations underline the need for dedicated processes for invited individuals without digital IDs or with limited language proficiency or digital literacy, to avoid making access to rare disease research dependent on the use of specific digital tools.

### Recommendations for improvement

To optimize eConsent processes in rare disease research, several strategies should be considered:

1. *Enhancing usability:* The complexity of digital consent systems can be a barrier for some participants, especially elderly and others with limited digital literacy and lack of access to digital solutions. Developing step-by-step guides with visuals and simplifying the login process can make the system more user-friendly, ensuring that participants can easily navigate through consent procedures without feeling overwhelmed.
2. *Improving communication:* Tailoring study materials to ensure clarity about the purpose, procedures, risks, and benefits of the research is crucial. For rare disease studies, simplifying complex medical language and using clear, concise messaging can help participants better understand their role in the research and how it might benefit both them and others with the same condition.
3. *Strengthening data accuracy:* Ensuring that participant data is accurate is critical for both research integrity and ethical compliance. Implementing automatic checks for duplicate registrations and deceased participants can reduce errors and ensure that consent remains valid and up to date.
4. *Facilitating long-term data sharing:* The rare disease community relies on long-term data sharing to accelerate research. eConsent systems should enable participants to manage their consent preferences over time, allowing them to update or withdraw consent if their views or circumstances change.
5. *Addressing language and cultural barriers:* For multilingual and multicultural populations, eConsent systems should offer translated materials. This is particularly important in rare disease research where excluding non-native speakers may further exacerbate existing inequities.
6. *Combining eConsent with optional in-person or virtual appointments:* While eConsent can simplify logistics and increase geographic reach, some participants, such as families of children with complex needs, individuals with limited digital literacy and older participants, may benefit from interacting face-to face with a healthcare professional or study team member. Offering optional in-person, telephone, or video-based appointments alongside the electronic platform has the potential to ensure that questions are addressed, comprehension is improved, and that the consent process remains ethically robust.

## Conclusion

In conclusion, electronic consent systems hold significant promise for improving the informed consent process, particularly in rare disease research. By making the process more accessible, transparent, and user-friendly, eConsent platforms can enhance participant understanding, encourage data sharing, and streamline administrative tasks. The recommendations discussed, i.e. enhancing usability, improving communication, and strengthening data accuracy, are essential steps in ensuring that the consent process is ethical, robust and effective. This study offers valuable insights for optimizing eConsent processes, with implications not only for genomic research in rare diseases but also for broader adoption in clinical and epidemiological studies**.**

## Supplementary Information

Below is the link to the electronic supplementary material.

Supplementary Material 1

## Data Availability

All data is available in the article files.

## Abbreviations

APA: American psychological association
API: Application programming interface
BankID: Swedish electronic identification system
CIOMS: Council for International Organizations of Medical Sciences
eConsent: Electronic informed consent
ERN: European reference network
ERN-ITHACA: European reference network on rare congenital malformations and rare intellectual disability
FDA CRF 21 Part 11: U.S. food and drug administration code of federal regulations title 21 part 11
GAMP 5: Good automated manufacturing practice version 5
GMC: Genomic medicine center
GMS: Genomic medicine Sweden
GMS-RD: Genomic medicine Sweden—rare diseases
ICH E6: International council for harmonisation guideline for good clinical practice E6
ID: Intellectual disability
NGP: National genomic data platform
UDN Sweden: Undiagnosed diseases network Sweden
VUS: Variant of uncertain significance
WGS: Whole genome sequencing
