# Supplementary material for: Implementing electronic informed consent in rare disease genomics
Source: Sci Rep. 2025 Dec 23;15:44419. doi: 10.1038/s41598-025-32740-1 (PMC12738699; doi:10.1038/s41598-025-32740-1)
Supplement: Supplementary file 1 — Supplementary Material 1 [file 41598_2025_32740_MOESM1_ESM.docx]

# Supplementary material

## Examples of letter templates sent to patients/families (translated from Swedish)

Letter template: GMC-K adult singleton

We are reaching out to you because you previously provided a sample for genetic analysis as part of a genetic investigation. Your sample has been analyzed using a method called Whole Genome Sequencing. This analysis generates a large amount of data that we would like to further process and analyze. This work is conducted as part of a research project within a larger study called GMS-RD (Genomic Medicine Sweden–Rare Diseases).

Our hope is that this project will contribute to improved diagnostics and a better understanding of the role of genetics in the development of various diseases.

To participate in the project, we need your consent. You can easily provide your consent by logging into Min forskning and signing with your BankID.

To give your electronic consent to participate in the study, please follow these steps:

1. Visit https://minforskning.se/study/gms-rd

2. Under the "Start" section, you will find basic information about the study.

3. Scroll down and select the healthcare unit: GMC-K (Karolinska University Hospital).

4. Click on GMC-K, then log in with BankID on Min forskning at the bottom of the page.

5. Read through the participant information, create a personal profile, and sign using BankID.

You are always welcome to contact us if you have any questions.

Phone: xxx

Letter template: GMC South, pediatric trio

I am writing to you since you and your child have previously submitted a sample for genetic analysis of your DNA within the healthcare system (whole genome sequencing, WGS).

You are hereby invited to participate in a research study called *GMS-RD: Identification of Novel Causes of Rare Genetic Diseases*. This study aims to identify new genetic causes of disease in individuals with rare diagnoses (conditions that affect only a small number of people) and to investigate these disorders. One of the main goals of the study is to increase the proportion of patients who receive a genetic diagnosis.

In order to conduct research on the samples from you and your child, we need your consent, and we currently have no record of your signature. Only adults can consent to participate in a research study. As the legal guardian, you therefore need to consent on behalf of your child, signing both for yourself and for your child. If the child has two legal guardians, the other guardian also needs to provide consent for the child.

To provide electronic consent to participate in the study, please follow these steps:

1. Open a web browser and go to the website: <https://minforskning.se/study/gms-rd>. We recommend using a computer, but a smartphone or tablet also works.
2. Read about the study under the “Start” section.
3. If you wish to participate in the study, click on “Read more and join”.
4. The first time you visit the website, you as an adult will need to create a personal profile on minforskning.se before you can proceed with the consent process for yourself and your child.
5. On the “Read more and join” page, you can choose between “Consent for yourself” or “Consent as legal guardian”.
6. If you choose “Consent as legal guardian”, a list of all children for whom you have legal custody will be shown. Select the child who has undergone a genetic investigation and for whom you wish to give consent.
7. Then select the healthcare provider “GMC South (Skåne University Hospital)”.
8. You can then read the participant information and sign the consent using BankID.
9. Repeat from step 5 if you also want to give consent for yourself or for another child.

Please feel free to contact me at any time (email: xxx or phone: xxx) if you have any questions or if you would like to discuss this further!
